# Cognitive Assessment Tools for Screening Older Adults With Low Levels of Education: A Critical Review

**DOI:** 10.3389/fpsyt.2019.00878

**Published:** 2019-12-13

**Authors:** José Wagner Leonel Tavares-Júnior, Ana Célia Caetano de Souza, Gilberto Sousa Alves, Janine de Carvalho Bonfadini, José Ibiapina Siqueira-Neto, Pedro Braga-Neto

**Affiliations:** ^1^Department of Clinical Medicine, Division of Neurology, Universidade Federal do Ceará, Fortaleza, Brazil; ^2^Center of Health Sciences, Universidade Estadual do Ceará, Fortaleza, Brazil; ^3^Neurology Service, Hospital Universitário Walter Cantídio, Universidade Federal do Ceará, Fortaleza, Brazil; ^4^Translational Psychiatry Research Group, Universidade Federal do Maranhão, São Luís, Brazil

**Keywords:** mental status tests, dementia tests, literacy, educational status, mild cognitive impairment

## Abstract

**Introduction:** Cognitive assessment of older adults who are either illiterate or with low levels of education is particularly challenging because several battery tasks require a certain educational background. Early detection of mild cognitive impairment (MCI) in the elderly using validated screening tools is of great importance since this population group could benefit from new drugs that are being investigated for the treatment of dementias. Cutoff scores for psychometric properties of cognitive tests are not well established among adults with low levels of education. The present study aimed to critically review the literature on cognitive assessment tools for screening cognitive syndromes including MCI and Alzheimer’s disease (AD) in older adults with low levels of education.

**Methods:** We conducted a systematic search of MEDLINE, LILACS, Cochrane, and SCOPUS electronic databases of cross-sectional and prospective studies with adults over 55 years of age.

**Results:** We found a significant number of assessment tools available (n = 44), but only a few of them showed diagnostic accuracy for the diagnosis of MCI and AD in older adults with low levels of education: the Mini-Mental State Exam; the Montreal Cognitive Assessment; the Persian Test of Elderly for Assessment of Cognition and Executive Function; the Six-Item Screener; and the Memory Alteration Test. Few studies evaluated individuals with low levels of education, with a wide range of cutoff scores and cognitive test batteries.

**Conclusion:** We found that a small number of studies evaluated adults with 4 years of formal education or less. Our findings further support the importance of developing specific tools for the assessment of older adults with low levels of education.

## Introduction

Dementia is characterized by cognitive impairment and loss of function (1). The growth of population aging over the past few decades has been associated with an increase in cognitive disorders. Data from Alzheimer’s Disease International (ADI) shows there were 46.8 million people living with dementia worldwide in 2015, and it is estimated this number will be 74.5 million in 2030 and 131.5 million in 2050. Alzheimer’s disease (AD) is the most common form of dementia and accounts for 50–70% of dementia cases. There were an estimated 26 million people living with AD in 2015 and it is believed there will be as many as 41 million by 2030 and 72 million by 2050 (1).

Mild cognitive impairment (MCI) is the intermediate stage between cognitive decline of healthy aging and dementia (2). The prevalence of MCI is 12–18% among adults over 65 years of age and the annual progression rates from MCI to AD are 10–15% (3, 4). Early detection of MCI in the elderly using validated screening tools is of great importance since this population group could benefit from new drugs that are being investigated for the treatment of neurodegenerative diseases including AD. Furthermore, evidence shows that, since MCI is a transitional phase between normal aging and AD, there is less brain involvement and those affected are more likely to benefit from drug therapies (5, 6).

Cognitive assessment tools are commonly used for screening impairment, differential diagnosis, determining disease severity, as well as monitoring disease progression in patients (7). A major challenge for the initial assessment of age-related cognitive changes is to find a screening tool that is both sensitive and specific for differential diagnosis of cognitive impairment. Both ceiling effects and floor effects limit the ability of a test or some of its items to accurately assess cognitive decline (8). These effects have been reported in several studies and they are primarily related to educational background (9). The ceiling effect occurs when score distribution is skewed and a measurement is determined by the proportion of people scoring at the high end, thereby preventing to detect health improvements. The opposite is the floor effect that occurs when a measurement is determined by the proportion of people scoring at the low end, thereby preventing to detect health declines (10).

Another important aspect is to have available free, easy-to-use assessment tools that do not require specialized training and have the ability to accurately discriminate cognitive decline in adults with normal aging, MCI, and dementia (4). Cognitive assessment of older adults who are either illiterate or with low levels of education is particularly challenging because several battery tasks require a certain educational background (11–13). There are an estimated 758 million illiterate adults in the world (11) and 13 million people are estimated to be illiterate in Brazil (11). Prospective cohort studies have shown an association between low education and higher risk of developing AD (14–17). Yet, few studies have examined the performance of cognitive assessment tools in adults with low education.

Cutoff scores for psychometric properties of cognitive tests are not well established among adults with low levels of education. Furthermore, there is a scarcity of studies evaluating assessment tools for screening older adults with low levels of education. A better understanding of the accuracy of different cognitive batteries is crucial for early diagnosis and intervention, and epidemiological studies are needed to further explore how education background affects an individual’s performance on different cognitive dimensions.

The present study aimed to critically review the literature on cognitive assessment tools for screening cognitive syndromes including MCI and AD in older adults with low levels of education.

## Methods

An integrative literature review was conducted to gather and summarize the body of evidence available from original articles. This integrative review study included six stages: Step 1—formulation of the central research question (theme identification); Step 2—definition of inclusion and exclusion criteria and literature search; Step 3—categorization of primary studies (definition of data to be extracted from the selected studies); Step 4—assessment of the studies included; Step 5—interpretation of results; Step 6—knowledge synthesis of the results obtained from the studies assessed (18–20).

The central research question was formulated using the PVO method where P is the study population (adults over 55 years of age with low education, i.e., 4 years of formal education or less); V is the variable (cognitive assessment tools); and O is the outcome (MCI and AD).

The guiding question of our review was: “Which assessment tools are used for cognitive screening of **MCI or AD** in older adults with low levels of education?” The inclusion criteria were English language articles in the electronic databases Medical Literature Analysis and Retrieval System Online (MEDLINE), Latin American and Caribbean Health Sciences Literature (LILACS), Cochrane, and SCOPUS; cross-sectional or prospective design; outpatient or population-based samples of adults over 55 years of age with low education (4 years of education or less); and **assessment of the use of cognitive tools for MCI or AD diagnosis**. We did not search the following information sources for this review: guidelines; institutional protocols; self-administered or telephone-based cognitive assessment instruments; and studies that used cognitive assessment instruments for diagnosing other psychiatric or neurological conditions. The publications were individually searched and selected by two investigators during June and July 2019.

**Figure 1:**
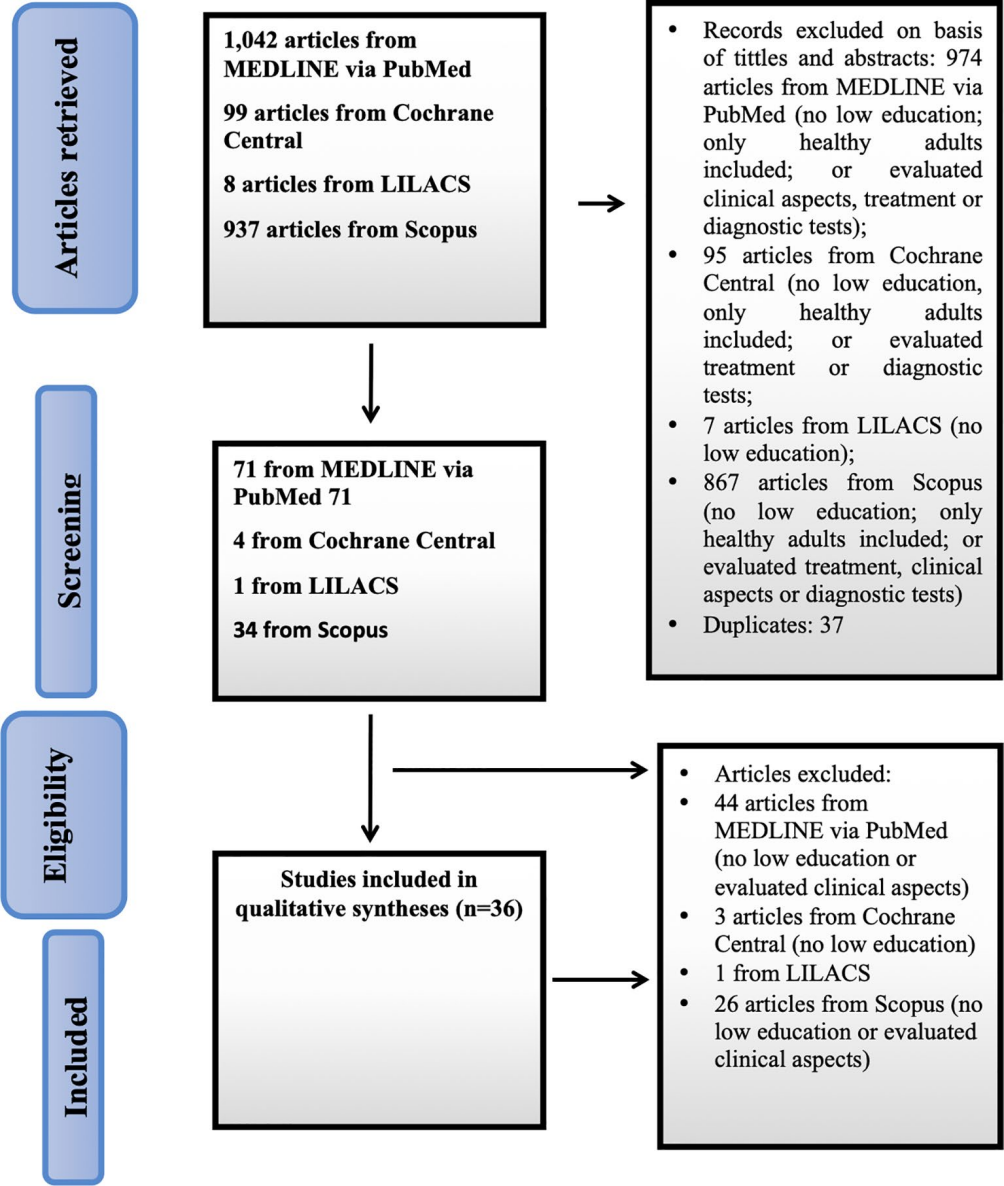
Flowchart of study selection.

The Preferred Reporting Items for Systematic Review and Meta-Analyses (PRISMA) guidelines (21) were used as a basis for the search and selection of studies (**Figure 1**). A questionnaire was developed to help data extraction (22). Two matrices were constructed to present the results: the first one included study characteristics and the second one included cognitive tools and main results reported.

A search strategy was created to conduct searches in the following databases: MEDLINE *via* PubMed from the US National Library of Medicine; LILACS; Cochrane; and SCOPUS *via Coordenação de Aperfeiçoamento de Pessoal de Nível Superior* (CAPES) with no time restriction. To expand our search, we chose to use natural controlled language. The following descriptors (bold), synonyms, natural language, and Boolean operators were used to cross-check the databases: MEDLINE (Medical Subject Headings [MeSH]: **search strategy**—*(aged or elderly or old or elder) and (literacy or illiteracy or education or “low education”) and (“mental status and dementia tests” or tool or instrument or status or test) and (“Alzheimer disease” or alzheimer´s) and (“mild cognitive impairment” or “cognitive dysfunction”).*

To minimize selection bias (misinterpretation of results and study design), the literature search and data extraction were conducted by two investigators independently and any discrepancies were resolved by consensus.

## Results

**Figure 1** shows the flowchart of the study selection process according to the PRISMA guidelines. A total of 2,086 articles were retrieved and read. Thirty-six studies were selected for our review.

**Table 1** describes the studies assessed. The sample sizes ranged from 50 to 10,432 participants. The studies were conducted in 17 countries, and most of them (13.88%) were from China and Spain.

**Table 1:** Articles included in the integrative literature review.

Authors, Year	Country	Study Design	Primary Study Objective	Level of Education	Sample Size	Participant Age	Cognitive Assessment Tool used	Main Results
Sun Y et al., 2014 (23)	Taiwan	Cross-sectional	To assess prevalence of MCI and dementia	Illiterate 32.1% ≤6 years 45.2% > 6 years 22.6%	10,432 (dementia 929, MCI 2,049, indeterminate 419)	Mean age (SD) 76.2 years (6.7)	MMSE Taiwan version	Prevalences: dementia 8.04% MCI 18.76%
Kim KW et al., 2011 (24)	South Korea	Cross-sectional	To assess prevalence of MCI and dementia in adults over 65 years of age	Illiterate 31.3% 1–6 years 38.1% > 7 years 30.6%	6,141	65–69 years 32.2% > 70 years 67.8%	CERAD-Korean version, Clinical Assessment Battery	Prevalences: dementia 8.1%, AD 5.7%, MCI 24.1%
Chen MR et al., 2010 (25)	China	Cross-sectional	To validate SIS for quick detection of cognitive impairment	Illiterate 7.2% 1–6 years 16.4% > 6 years 76.3%	1,976 (healthy aging 475, MCI 440, AD 1,061)	Mean age (SD) 71.87 years (8.71)	SIS, MMSE	SIS: MCI Sn 34.3%, Sp 90.1%, MMSE: cutoff score <4 years of schooling ≤17; Sn 94.3%; Sp 95.0%
Chang J et al., 2014 (26)	Hong Kong	Cross-sectional	To assess the effect of education on tools for screening older population	Mean schooling 4.7 years (SD 4.6; 0–20)	788 (AD 405, controls 383)	Mean age (SD) 72.08 years (7.27)	MMSE, ADAS-Cog, Verbal Fluency, Abstract Thinking, and Visual/DS	Effect of educational background on MMSE, language sub-item
O’Bryant SE et al., 2013 (27)	United States	Cross-sectional	To characterize a Mexican American population with MCI and AD	Mean schooling (SD) AD 5.9 years (4.5) MCI 6.6 years (4.2) Controls 8.1 years (4.2)	1.069 non-Hispanic white (*n* = 633: AD 160, MCI 97, controls 376) Mexican American (*n* = 436, AD 35, MCI 67, controls 337)	Mean age (SD) AD 73.6 years (9.1) MCI 61.9 years (12.3) Controls 58.7 years (9.9)	MMSE	Mean MMSE score (SD) AD 18.5 (5.0) MCI 24.7 (3.6) Controls 27.5 (2.8)
de Paula JJ et al., 2013 (28)	Brazil	Cross-sectional	To validate an unstructured neuropsychological assessment tool for clinical use	Mean schooling (SD) MCI 4.71 years (4.00) AD 4.82 years (3.46) Controls 5.22 years (4.29)	274 (96 controls, MCI 85, AD 93)	Mean age (SD) Controls 72.61 years (7.76), MCI 73.18 years (8.46), AD 74.57 years (6.65)	RAVLT, FAB, verbal fluency, SDT, CDT, DS, TT and TN-LIN	Study protocol: Sn >70%, Sp >70% for AD and MCI
Sánchez Benevides G et al., 2014 (29)	Spain	Cross-sectional	To evaluate neuropsychological assessment tool for MCI and AD	Mean schooling (SD) MCI 8.0 years (4.7) AD 7.6 years (4.6) Controls 10.4 years (5.4)	535 (controls 356, MCI 79, AD 100)	Mean age (SD) Controls 64.9 years (9.3) MCI 72.8 years (6.5) AD 74.7 years (7.5)	MMSE, DS, WAIS, TMT, SDMT, BNT, TT, SVOSPB, JLO, verbal fluency, ROCF, FCSRT, phonemic fluency, SCWIT, TLDU	Mean MMSE scores (SD) Controls 28.7 (1.5) MCI 25.7 (2.2) AD 20.2 (4.0) FCSRT showed best diagnostic accuracy for AD vs. controls
Mellor D et al., 2016 (30)	China	Cross-sectional	To assess effectiveness for discriminating MCI or AD vs. healthy controls	Mean schooling (SD) MCI 5.17 (4.78) AD 3.72 (4.14)	1.027 (controls 708, AD 267, MCI 50)	Mean age (SD) 72.54 years (8.40)	MMSE Chinese version, MoCA	(cutoff score, Sn, Sp) MCI: MMSE 25.50/68/83 MoCA 22.50/.87/73
Javadi PSHS et al., 2015 (31)	Iran	Cross-sectional	To characterize illiterate and literate older adults; PEACE scale cutoff scores for AD	Different levels of education—illiterate and literate	101 (controls 33, MCI 30, AD 38)	Mean age (SD) AD 74.60 years (8.02) MCI 72.5 years (7.2) Controls 67.84 years (7.29)	PEACE, GPCOG, FAST, MMSE, WMS	MMSE < 4 years of schooling MCI 18.75 (1.75) AD 12.64 (3.78) PEACE AD 67.5 (Sn 75.8%, Sp 97.4%)
Chong MS et al., 2010 (32)	Singapore	Cross-sectional	To compare FAB-X and MMSE for screening early cognitive impairment	Mean schooling (SD) MCI 7,1 years (4.4) Controls 9.6 years (4.4)	180 (controls 100, MCI 21, dementia 59)	Mean age (SD) Controls 63.7 years (6.51) MCI 69.3 years (7.91)	FAB Chinese version, MMSE, verbal fluency, BNT, WAIS	FAB MCI and mild dementia Cutoff scores 12/13 (Sn 92%; Sp 78.7%) MMSE (Sn 77%; Sp 91.2%) More effective when combined
Ng A et al., 2013 (33)	Singapore	Cross-sectional	To assess MoCA diagnostic accuracy for MCI and AD in older adults	Mean schooling (SD) MCI 10.93 years (4.28) AD 6.97 years (4.47) Controls 12.07 years (3.20)	212 (controls 103, MCI 49, AD 60)	Mean age 62.35 years	MMSE, MoCA	MoCA cutoff scores MCI <26 (≤10 years of schooling); < 27 (> 10 years of schooling), Sn >94%
Saka E et al., 2006 (34)	Turkey	Cross-sectional	To assess performance of ECR for discriminating dementia, AD and MCI vs. controls	Mean schooling (SD) MCI 8.4 years (5.0) AD 6.7 years (4.9) Controls 8.4 years (4.9)	113 (MCI 80, controls 33)	Mean age (SD) MCI 69.4 years (8.3) AD 73.8 years (6.1) Controls 72.7 years (6.7)	MMSE, ECR	ECR showed good performance to discriminate AD vs. controls (AUC 0.990), moderate performance for MCI vs. controls (AUC 0.625)
Tognoni G et al., 2005 (35)	Italy	Cross-sectional	To assess prevalence of dementia in older population	< 4 years (15.8%), > 4 years (84.2%)	1.600 (AD 68, MCI 149)	Mean age (SD) 74.65 years (7.26)	MMSE, CAMDEX	Prevalence of amnesic MCI 4.9%, AD 4.2%
Afgin AE et al., 2012 (36)	Israel	Cross-sectional	To assess prevalence of MCI and AD and conversion rate from MCI to AD within a year or more	0 years 51% 1–4 years 23% 5–8 years 21% > 8 years 5%	944 (controls 497, MCI 303, SD 13, VD 39, AD 92)	Mean age (years) (SD) DA 78.5 (7.7), MCI 72.8 (6.1), Controls 70.7 (5.5) by age groups (%) 65–69 (39.4%) 70–79 (46.4%) ≥80 (14.1%)	MMSE Arabic version, Brookdale Cognitive Screening Test	Prevalence AD 9.8%, MCI 32.1% MMSE <4 years of schooling cutoff scores (SD) MCI illiterate 17.8 (1.9), 1–4 years of schooling 19.6 (3.4);
Chaves ML et al., 2009 (37)	Brazil	Cohort	To assess incidence rate of AD and MCI in a community of older adults	Mean schooling (SD) 9.06 years (5.50)	345 (no details available)	Mean age (SD) 70.37 years (7.15)	MMSE	Incidence rate per 1,000 MCI 13.2 AD 14.8
Custodio N et al., 2017 (38)	Peru	Cross-sectional	MAT performance to discriminate controls, MCI and AD in adults with low education	Mean schooling (SD) AD 2.65 years (1.28) MCI 2.53 years (1.46) Controls 2.57 years (1.45)	247 (controls 121, AD 81, MCI 45)	Mean age (SD) AD 74.18 years (3.81) MCI 71.09 years (4.20) Controls 69.53 years (4.11)	MAT, RAVLT, WMS, TMT A and B, ROCF, BNT, WCST, DS, WAIS, MMSE	MAT AD vs. MCI AUC 99.60% MCI vs. controls AUC 99.56% Mean MMSE scores (SD): MCI 21.36 (0.98); MAT MCI 30.53 (2.54)
Ravaglia G et al., 2008 (39)	Italy	Cohort	To assess incidence and prevalence rates of MCI in older adults	Mean schooling (SD) 4.3 years (2.3)	1.016 (controls 865, MCI 75, dementia 60, indeterminate 19)	Mean age (SD) Controls 73.6 years (6.1) MCI 78.1 years (8.3)	MMSE, Mental Deterioration Battery	MCI prevalence 7.7%; incidence rate in 4 years 76.8 per 1,000 persons-years
Rahman TTA et al., 2009 (40)	Egypt	Cross-sectional	To assess validity of MoCA version to detect MCI	Mean schooling MCI 8.2 years (5.5)	184 (MCI 94, controls 90)	Mean age (SD) 64.5 years (6.8)	MoCA Arabic version, CAMCOG	MoCA MCI Sn 92.3% Sp 85.7%
Freitas S et al., 2013 (41)	Portugal	Cross-sectional	To validate MoCA for screening of MCI and AD	Mean schooling (SD) MCI 6.50 years (4.56) AD 6.2 years (4.11) Controls 6.39 years (4.30)	360 (MCI 90, AD 90, controls 180)	Mean age (SD) 71.86 years (7.895)	MMSE, MoCA	Cutoff scores/Sn/Sp MCI MoCA <22/81/77, MMSE <29/67/72
Borson S et al., 2005 (42)	United States	Cross-sectional	To compare the Mini-Cog Test vs. MMSE for screening cognitive impairment	Literate 76%, Semi-literate and illiterate 24%	371 (controls 140, AD 112, MCI 71, other dementias 48)	Mean age Controls 73 years MCI 74 years AD 78 years	CASI, MMSE, Mini-Cog	AD and MCI diagnostic accuracy Mini-Cog 83% MMSE 81%
Boycheva E et al., 2018 (43)	Spain	Cross-sectional	To assess clinical performance of MDRS for screening MCI and AD in older adults	Mean schooling (SD) 7.08 years (3.57)	125 (AD 45, MCI 37, controls 43)	Mean age 75.12 years (6.83)	MDRS, MMSE, WAT, FCSRT, BNT, SCWIT, GSBT, verbal fluency, phonemic fluency, WAIS	MDRS-2 cutoff score MCI vs. controls 131 (Sn 89%, Sp 81%)
Pezzotti P et al., 2008 (44)	Italy	Cross-sectional	To compare agreement of MMSE between primary care and specialist practitioners	0–5 years 68.1% > 5 years 31.9%	317 (MCI 40, AD 95, other dementias 98, healthy controls 84)	Mean age not available	MMSE, Mental Deterioration Battery, SCWIT	Mean MMSE score Primary care providers 15.8 Specialists 17.4 (kappa 0.86)
Julayanont P et al., 2015 (45)	Thailand	Cross-sectional	To assess validity of MoCA-B for MCI in a population with low education	Mean schooling (SD) MCI 2.9 years (1.7)	85 (controls 43, MCI 42)	Mean age (SD) MCI 70.2 years (6.6)	MoCA-B, MMSE Thai version	Mean score (SD) MMSE MCI 18.9 (3.0) MoCA-B 21.3 (3.8)
Matías-Guiu JA et al., 2017 (46)	Spain	Cross-sectional	To validate LASSI-L scale for MCI and AD diagnosis	Mean schooling (SD) Controls 8.52 years (4.98), MCI 7.61 years (4.79) AD 7.06 years (4.20)	164 (controls 97, MCI 34, AD 33)	Mean age (SD) 73.4 years (10.0)	LASSI-L, MMSE	LASSI-L discrimination AD vs. controls AUC 0.986; MCI vs. controls AUC 0.909
Chu LW et al., 2015 (47)	Hong Kong	Cross-sectional	To validate MoCA Chinese version for screening MCI and AD in older adults	Mean schooling (SD) Controls 6.97 years (4.69) MCI 4.62 years (5.19) AD 4.56 years (5.00)	266 (controls 115, MCI 87, AD 64)	Mean age 75.3 years	MoCA Cantonese Chinese version, MMSE Chinese version	MoCA cutoff score/Sn/Sp MCI 22–3/78%/73%, AD 19–20/94%/92%
Kurt P et al., 2014 (48)	Turkey	Cross-sectional	To develop a composite score for DEKOD in an older population	< 4 years of schooling 45.7% > 5 years of schooling 54.3%	444 (controls 338, dementia 53, MCI 53)	Mean age (SD) controls 70.7 years (5.4) dementia 74 years (7.8) MCI 71.7 years (5.6)	DEKOD, MMSE Turkish version	MMSE <4 years of schooling: cutoff score/Sn/Sp Dementia 17–18/95%/83% MCI 22–23/67%/55% DEKOD Dementia 49–50/91%/90% MCI 60–61/70%/65%
Bae JB et al., 2015 (49)	South Korea	Cross-sectional	To assess incidence rates of AD and MCI within 3.5 years of follow-up	Mean schooling (SD) 6.8 years (5.4)	181 (not available)	Mean age 71.7 years	MMSE, Korean CERAD, Clinical Assessment Battery, CERAD-K-N	Incidence rate per 1,000 persons-years AD 7.9 MCI 28.1
Paddick SM et al., 2015 (50)	Tanzania	Cohort	To assess outcomes, prevalence and profiles of patients with MCI in a rural community	Illiterate with MCI 55.5% Literate (at least elementary schooling) 44.5%	296 (MCI 46, dementia 78, controls 172)	Mean age 82 years	CERAD	Prevalence of MCI 7%
Choi SJ et al., 2008 (51)	South Korea	Cross-sectional	To assess prevalence of AD and MCI in older Korean adults	Mean schooling (SD) MCI 4.93 years (3.27) DA 1.69 years (3.09)	175 (controls 102, MCI 57, AD 16)	Mean age (SD) 74.3 years (16.7)	Korean MMSE, CERAD-K	Prevalences: AD 9.0% MCI 32.9%
Gavrila D et al., 2009 (52)	Spain	Cross-sectional	To assess prevalence of MCI and dementia in older adults	Illiterate 7.8% Less than elementary 19.7%, Elementary or more 72.5%	1,017 (controls 726, MCI 235, AD 30, other dementias 26)	Mean age (SD) 73.9 years (6.8)	MMSE, CAMDEX, Blessed Dementia Scale	Prevalence of amnesic MCI 8.7%
Wang Bet al., 2011 (53)	China	Cross-sectional	To describe clinical characteristics of patients in a memory center	0 years 8% 1–5 years 15% > 6 years 77%	2,789 (healthy aging 604, MCI 635, AD 1084, other diagnoses 466)	< 50 years 5.2% 50–59 years 14.9% > 60 years 79.9%	MMSE	Population distribution in a memory center: AD 83.7% MCI 22.8%
Zhou Y et al., 2015 (54)	United States	Cross-sectional	How to adjust MoCA for educational background in a Spanish-speaking population	Mean schooling (SD) Controls 10.3 years (6.4) MCI 7.1 years (4.8) Dementia 6.8 years (5.5)	50 (AD 18, MCI 21, controls 6, other dementias 5)	Mean age (SD) 71.4 years (9.7)	MoCA	It was required to adjust scores 3–4 points for those <6 years of schooling
Chen K et al., 2016 (55)	China	Cross-sectional	To assess performance of MoCA-B Chinese version for screening MCI in older adults	Groups by years of schooling (< 6, 7–12, > 12). Mean schooling (SD) of those with <6 years: controls 4.8 years (1.7), MCI 3.3 years (2.4), AD 3.7 years (2.5)	704 (MCI 264, AD 160, controls 280)	Mean age (SD) of those with <6 years of schooling controls 68.2 years (9.1) MCI 68.5 years (8.5) AD 67.9 years (9.4)	MMSE, AVLT, ROCF, BNT, Verbal Fluency, TMT, SCWIT, SDMT, MoCA-B Chinese version	Cutoff scores Sn/Sp of those with <6 years of schooling: MCI MoCA-BC 19/87.9/81.0 MMSE 26/86.2/60.3
Matías-Guiu JA et al., 2017 (56)	Spain	Cross-sectional	To compare diagnostic properties of five cognitive screening tools	Mean schooling (SD) Controls 8.01 years (5.40) AD 7.10 years (4.07)	160 (AD 92, controls 68)	60–69 years 7.5% 70–79 years 48.8% 80–93 years 43.8%	MMSE, ACE-III, MIS, MoCA, RUDAS	AD: ACE-III AUC 0.897; RUDAS AUC 0.889; MMSE AUC 0.874; MIS AUC 0.866; MoCA AUC 0.856
Huang YY et al., 2019 (57)	China	Cross-sectional	To compare diagnostic accuracy of MoCA-B and MoCA-Beijing for screening MCI in people with different levels of education	No supplementary table available	808 (MCI 295, AD 254, controls 259)	Not available	MoCA, MMSE, AVLT; ROCF, BNT, Verbal Fluency, TMT, SCWIT, SDMT	Performance in discriminating MCI vs. controls: MoCA B AUC 0.95 MoCA-BJ AUC 0.87
Khedr E et al., 2015 (58)	Egypt	Cross-sectional	To assess prevalence of MCI and dementia in Egyptian adults over 60 years of age	MCI 75% illiterate AD 75% illiterate	691 (MCI 12, dementia 35)	Mean age (SD) MCI 67.3 years (7.1), AD 69.3 years (7.7)	*MMSE, WMS	MCI prevalence 1.72/100

MMSE, Mini-Mental State Examination; BNT, Boston Naming Test; AVLT, Auditory Verbal Learning Test; MoCA, Montreal Cognitive Assessment; MoCA B, MoCA Basic; ACE, Addenbrooke Cognitive Examination; TMSE, Taiwanese Mental State Examination; CERAD, Consortium to Establish a Registry for Alzheimer’s Disease Assessment Packet; SDT, Stick Design Test; TT, Token Test; SDMT, Symbol Digit Modalities Test; SVOSPB, Selected test of the Visual Object and Space Perception Battery; JLO, Judgment of Line Orientation; TLDU, Tower of London Drexel University version; WAT, Word Accentuation Test; GSBT, Gesture Sequences subtest from the Barcelona Test; SIS, Six-Item Screener; ADAS-Cog, Alzheimer’s Disease Assessment Scale’s cognitive subscale; RAVLT, Rey-Auditory Verbal Learning Test; FAB, Frontal Assessment Battery; CDT, Clock Drawing Test; DS, Digit Span; TMT, Trail Making Test; ROCF, Rey-Osterrieth Complex Figure; FCSRT, Free and Cued Selective Reminding Test; SCWIT, Stroop Color-Word Interference Test; PEACE, Persian Test of Elderly for Assessment of Cognition and Executive function; GPCOG, General Practitioner assessment of Cognition; FAST, Functional Assessment Staging; WMS, Wechsler Memory scale; ECR, Enhanced cued recall test; CAMDEX, Cambridge Examination for Mental Disorders of the Elderly; WAIS, Wechsler Adult Intelligence Scale–Revised; MDRS, Mattis Dementia Rating Scale; BDAE, Boston Diagnostic Aphasia Examination; RDT, Rosen Drawing Test; MAT, Memory Alteration Test; WCST, Wisconsin Card Sorting Test; CASI, Cognitive Abilities Screening Instrument; LASSI-L, Loewenstein-Acevedo Scale for Semantic Interference and Learning; DEKOD, Dokuz Eylül Kognitif Degerlendirme/Dokuz Eylul Cognitive Assessment; CERAD-K-N, CERAD-K Neuropsychological Assessment Battery; MIS, Memory Impairment Screen; AUC, Area Under the ROC Curve; CAMCOG, Cambridge Cognitive Examination; RUDAS, Rowland Universal Dementia Assessment Scale.

A wide range of cognitive assessment tools (n = 44) were used for MCI and AD diagnosis (**Table 1**). Of these, the *Mini-Mental State Examination* (*MMSE*) (59) was the most frequently used (86.11%), followed by the Montreal Cognitive Assessment (MoCA) (60) (27.77%).

The studies included in our review evaluated adults with different educational backgrounds. However, detailed information was not available in all studies. Adults with AD had 1.69 (51) to 7.6 years (29) of schooling and those with MCI had 2.53 (38) to 10.93 years (33) of schooling. The proportion of illiterate adults ranged from 1.8% (35) to 32.1% (23).

Most were cross-sectional studies (91.67%), followed by cohort studies (8.33%). Some of the studies (23, 24, 35, 36, 39, 50–52, 58) assessed the prevalence of MCI and AD ranging from 1.72% (58) to 32.9% (52) and 4.2% (35) to 9% (51), respectively. The number of adults with MCI ranged from 12 (58) to 2,049 (30) and the number of adults with AD ranged from 16 (51) to 1,061 (25). Mean age of the study participants ranged from 64.5 (40) to 82 years (50).

The Persian Test of Elderly for Assessment of Cognition and Executive Function (PEACE) was used in one study (28) to establish cutoff scores in individuals with AD. The sample consisted of 38 subjects with AD; some of them were illiterate (proportion not available). A cutoff score of 67.5 was set (sensitivity = 75.8%; specificity = 97.4%). The Six-Item Screener (SIS) was used in another study (25) that evaluated 440 individuals with MCI with a small proportion of individuals (< 25%) with low education (< 6 years of schooling). The SIS showed low sensitivity for screening MCI in this population (sensitivity = 34.3%; specificity = 90.1%). The Memory Alteration Test (MAT) was used in a single study (38) for discriminating MCI and AD from healthy individuals. The AUC of MAT to discriminate between early AD and amnestic mild cognitive impairment (aMCI) was 99.60% (sensitivity = 100.00%; specificity = 97.53%) and to discriminate between aMCI and controls was 99.56% (sensitivity = 99.17%; specificity = 91.11%). The mean score was 17.54 ± 4.67 for individuals with AD, 30.53 ± 2.54 for individuals with MCI and 41.97 ± 2.6 for healthy individuals. AD and MCI individuals and controls had on average 2.65 ± 1.28, 2.53 ± 1.46, and 2.57 ± 1.45 years of education, respectively.

In this review, six studies (**Table 2**) assessed cutoff scores of the MMSE for adults with 4 years of education or less (25, 36, 38, 40, 45, 48). MCI cutoff scores (SD) ranged from 17.8 (1.9) to 21.36 (0.98), but there was great variation in sensitivity and specificity. The Montreal Cognitive Assessment-*Basic (MoCA-B)* was evaluated in three studies (45, 55, 57). One of these studies established a cutoff score of 19 for detecting MCI, with 87.9% sensitivity and 81.0% specificity (55). Another one reported a cutoff score (SD) of 21.3 (3.8) for detecting MCI in adults with 4 years of education or less (45). Cutoff scores (SD) for AD ranged from 12.64 (3.78) to 18.32 (2.78) in these same studies. *Another* cognitive test battery reported was the Addenbrooke’s Cognitive *Examination* Revised (*ACE-R), which was used in* only one study but the cutoff score was not adjusted for low educational level. Mean ACE-R scores were 78.12 (12.79) for controls and 53.20 (14.76) for AD. This tool showed good diagnostic accuracy for diagnosing AD (AUC = 0.897) (25).

**Table 2:** Characteristics of studies that established cutoff scores for the MMSE and MoCA in adults with low education.

Authors, Year	Country	Study Design	Primary Study Objective	Level of Education	Sample Size	Participant Age	Cognitive Assessment Tool used	Main Results
Chen et al., 2010 (25)	China	Cross-sectional	To validate SIS for quick detection of cognitive impairment	Illiterate 7.2% 1–6 years 16.4% > 6 years 76.3%	1,976 (healthy aging 475, MCI 440, AD 1,061)	Mean age (SD) 71.87 years (8.71)	SIS, MMSE	SIS: AD Sn 88.5%, Sp 78.3%, MCI Sn 34.3%, Sp 90.1%, MMSE: cutoff score <4 years of schooling ≤17; Sn 94.3%; Sp 95.0%
Javadi PSHS et al., 2015 (31)	Iran	Cross-sectional	To characterize illiterate and literate older adults; PEACE scale cutoff scores for AD	Different levels of education—illiterate and literate	101 (controls 33, MCI 30, AD 38)	Mean age (SD) AD 74.60 years (8.02) MCI 72.5 years (7.2) Controls 67.84 years (7.29)	PEACE, GPCOG, FAST, MMSE, WMS	MMSE < 4 years of schooling MCI 18.75 (1.75) AD 12.64 (3.78) PEACE AD 67.5 (Sn 75.8%, Sp 97.4%)
Afgin AE et al., 2012 (36)	Israel	Cross-sectional	To assess prevalence of MCI and AD and conversion rate from MCI to AD within a year or more	0 years 51% 1–4 years 23% 5–8 years 21% > 8 years 5%	944 (controls 497, MCI 303, SD 13, VD 39, AD 92)	Mean age (years) (SD) DA 78.5 (7.7) MCI 72.8 (6.1) Controls 70.7 (5.5) by age groups (%) 65–69 (39.4%) 70–79 (46.4%) ≥80 (14.1%)	MMSE Arabic version, Brookdale Cognitive Screening Test	Prevalence AD 9.8%, MCI 32.1% MMSE <4 years of schooling cutoff scores (SD) MCI illiterate 17.8 (1.9), 1–4 years of schooling 19.6 (3.4); AD illiterate 12.7 (3.7), 1–4 years of schooling 12.6 (6.7)
Custodio N et al., 2017 (38)	Peru	Cross-sectional	MAT performance to discriminate controls, MCI and AD in adults with low education	Mean schooling (SD) AD 2.65 years (1.28) MCI 2.53 years (1.46) Controls 2.57 years (1.45)	247 (controls 121, AD 81, MCI 45)	Mean age (SD) AD 74.18 years (3.81) MCI 71.09 years (4.20) Controls 69.53 years (4.11)	MAT, RAVLT, WMS, TMT A and B, ROCF, BNT, WCST, DS, WAIS, MMSE	MAT AD vs. MCI AUC 99.60% MCI vs. controls AUC 99.56% Mean MMSE scores (SD): AD 18.32 (2.78); MCI 21.36 (0.98) MAT AD 17.54 (4.67); MCI 30.53 (2.54)
Julayanont P. et al., 2015 (45)	Thailand	Cross-sectional	To assess validity of MoCA-B for MCI in a population with low education	Mean schooling (SD) Controls 3.6 years (1.1) MCI 2.9 years (1.7)	85 (controls 43, MCI 42)	Mean age (SD) Controls 66.6 years (6.7) MCI 70.2 years (6.6)	MoCA-B, MMSE Thai version	MMSE <4 years of schooling—illiterate Mean score (SD) MMSE MCI 18.9 (3.0) MoCA-B 21.3 (3.8)
Kurt P et al., 2014 (48)	Turkey	Cross-sectional	To develop a composite score for DEKOD in an older population	< 4 years of schooling 45.7% > 5 years of schooling 54.3%	444 (controls 338, dementia 53, MCI 53)	Mean age (SD) controls 70.7 years (5.4) dementia 74 years (7.8) MCI 71.7 years (5.6)	DEKOD, MMSE Turkish version	MMSE <4 years of schooling: cutoff score/Sn/Sp Dementia 17–18/95%/83% MCI 22–23/67%/55% DEKOD Dementia 49–50/91%/90% MCI 60–61/70%/65%
Chen K et al., 2016 (55)	China	Cross-sectional	To assess performance of MoCA-B Chinese version for screening MCI in older adults	Groups by years of schooling (< 6, 7–12, > 12). Mean schooling (SD) of those with <6 years: controls 4.8 years (1.7), MCI 3.3 years (2.4), AD 3.7 years (2.5)	704 (MCI 264, AD 160, controls 280)	Mean age (SD) of those with <6 years of schooling controls 68.2 years (9.1) MCI 68.5 years (8.5) AD 67.9 years (9.4)	MMSE, AVLT, ROCF, BNT, Verbal Fluency, TMT, SCWIT, SDMT, MoCA-B Chinese version	Cutoff scores Sn/Sp of those with <6 years of schooling: MCI MoCA-BC 19/87.9/81.0 MMSE 26/86.2/60.3

SIS, Six-Item Screener; MMSE, Mini-Mental State Examination; PEACE, Persian Test of Elderly for Assessment of Cognition and Executive function; GPCOG, General Practitioner assessment of Cognition; FAST, Functional Assessment Staging; WMS, Wechsler Memory scale; MAT, Memory Alteration Test; RAVLT, Rey-Auditory Verbal Learning Test; TMT, Trail Making Test; BNT, Boston Naming Test; ROCF, Rey-Osterrieth Complex Figure; WCST, Wisconsin Card Sorting Test; DS, Digit Span; WAIS, Wechsler Adult Intelligence Scale–Revised; MoCA, Montreal Cognitive Assessment; MoCA B, MoCA Basic; DEKOD, Dokuz Eylül Kognitif Degerlendirme/Dokuz Eylul Cognitive Assessment; SCWIT, Stroop Color-Word Interference Test; AUC, Area Under the ROC Curve; AVLT, Auditory Verbal Learning Test; SDMT, Symbol Digit Modalities Test.

## Discussion

We carried out a critical review of cognitive assessment tools for screening cognitive syndromes in older adults with low levels of education. A significant number of assessment tools (n = 44) were used in the studies reviewed, but only a few of them showed diagnostic accuracy for the diagnosis of MCI and AD in adults with low education including MMSE, MoCA, PEACE, *SIS*, and MAT. The latter three were each used in one study only.

It is crucial to validate cognitive assessment instruments in populations with low education and to establish cutoff scores for screening these individuals in daily clinical practice. It would enable to monitoring healthy aging in such a quite large population (11) and evaluate older adults with low levels of education who are at risk of developing dementia syndromes (61). Besides, it could offer new insights to better understand the influence of education on cognitive reserve since there is a relationship between literacy and the functional organization of the human brain. Literacy acquisition improves early visual processing and phonological information processing (62). Indeed, functional neuroimaging studies have evidenced that large neural networks in both cerebral hemispheres have less functional connections in less educated individuals (63). Since there has been a move toward the development of *disease*-*modifying drugs* for AD, it will be paramount to have validated diagnostic tools for population-based assessments including older adults with low levels of education (64).

In agreement with literature reports, the MMSE was the most frequently used cognitive tool in the studies assessed (65). The MMSE is easy to administer and requires no specialized training and it has been validated in many countries (66). We assessed in our review a study that showed good agreement of the MMSE for cognitive screening (κ = 0.86) between primary care and specialist practitioners (44). However, studies *have* demonstrated the effect of *education on MMSE* scores. The MMSE has low sensitivity for MCI, does not perform well in assessing executive functions and has limiting floor and ceiling effects (9, 23, 40). A study conducted in Brazil has established MMSE cutoff scores of 20 for illiterate adults and 25 for those with 4 years of education or less (67).

The second most frequently used cognitive tool was the MoCA. The MoCA is a cognitive battery that includes tests sensitive to executive functions and has higher sensitivity for diagnosing MCI (24, 33, 68). However, MoCA scores are strongly influenced by educational background as MoCA tasks are designed for a certain level of education making it difficult to assess individuals who are either illiterate or with low levels of education (30). Therefore, a MoCA basic version (MoCA-B) was developed to include tasks designed to assess the same cognitive domain regardless of the level of education (45, 60, 68).

A systematic review of cognitive screening tools showed that the ACE-R is an outstanding test battery. It takes approximately 20 minutes to be administered and it includes tasks designed for different levels of schooling (52, 58). In our review, ACE-R cutoff scores for low education were not available.

The studies assessed included recommendations of specific cutoff scores and scales for diagnosis of MCI and AD in adults with low levels of education. One study using the MoCA-B suggested a cutoff score of 19 (55) for MCI diagnosis. For AD, one study showed a cutoff score of 23.5 (30) for the MMSE and another study suggested a cutoff score of 17 (41) for the MoCA.

The scarcity of cognitive assessment tests for evaluating adults with low levels of education is in line with the challenge of assessing cognitive function in individuals with low education (13). Individuals with low levels of education were excluded from many studies because there are no cutoff scores established for several assessment tools. Literacy acquisition increases performance in certain cognitive domains such as executive functions (14), improves visual processing, changes phonological codes, and strengthens the link between phonemic and graphic representation (62) However, to measure literacy through the number of years of formal education is not the most effective approach since there are so many different ways of learning even without attending school (12, 62).

This review study has some limitations that deserve mention. First, no information was available on the diagnostic accuracy of cognitive tools for MCI and AD. Furthermore, little information was available on the diagnostic accuracy of tools for assessing different MCI subtypes and stages of AD. Another caveat is that our search was limited to cognitive assessment tools that require a trained examiner and excluded self-administered and web-based screening tools. Besides, there is no consensus about the definition of low levels of education, which may partly explain heterogeneous results of the cognitive batteries evaluated. Lastly, the studies included this review applied various diagnostic criteria for AD, which prevented comparisons of results across them.

## Conclusions

The use of cognitive assessment tools that are easy to administer is still challenging given the high prevalence of low education in the global population. This review provides an overview of the most commonly used instruments for cognitive screening. We found that a small number of studies evaluated adults with 4 years of formal education or less and a wide range of cutoff scores for various cognitive test batteries. Our findings further support the need for the development of specific tools for assessing illiterate adults. Cognitive ability, formal logic, and abstract reasoning should be assessed as they could provide more accurate input for screening and interpretation of cognitive tests in older adults who are either illiterate or with low levels of education. Low-cost test batteries that are easy to administer should be validated because they can make a significant impact on screening of cognitive syndromes and enable early therapeutic interventions aimed at reducing morbidity and mortality of dementia. Further studies of test batteries adjusted to larger groups of adults with low levels of education and specific MCI subtypes and AD stages could help shed light on these points.

## Author Contributions

Conception and design of work: JT-J, AS, GA and PB-N. Acquisition, analysis or interpretation of data and work: JT-J, AS, PB-N and GA. Drafting the work: JT-J, AS, GA, JB, JS-N and PB-N. All authors were involved in critical revision of the manuscript for important intellectual content.

## Conflict of Interest

The authors declare that the research was conducted in the absence of any commercial or financial relationships that could be construed as a potential conflict of interest.
